# Management of post abortion complications in Botswana -The need for a standardized approach

**DOI:** 10.1371/journal.pone.0192438

**Published:** 2018-02-16

**Authors:** Tadele Melese, Dereje Habte, Billy M. Tsima, Keitshokile Dintle Mogobe, Mercy N. Nassali

**Affiliations:** 1 Department of Obstetrics and Gynecology, University of Botswana, Gaborone, Botswana; 2 Consultant Public Health Specialist, Addis Ababa, Ethiopia; 3 Department of Family Medicine and Public Health, University of Botswana, Gaborone, Botswana; 4 School of Nursing, University of Botswana, Gaborone, Botswana; National Academy of Medical Sciences, NEPAL

## Abstract

**Background:**

Post abortion complications are the third leading cause of maternal death after hemorrhage and hypertension in Botswana where abortion is not legalized. This study aimed at assessing the management of post abortion complications in Botswana.

**Methods:**

A retrospective study was conducted at four hospitals in Botswana in 2014. Socio-demographic, patient management and outcomes data were extracted from patients’ medical records. Descriptive statistics and chi-square test were used to analyze and present the data.

**Result:**

A total of 619 patients’ medical records were reviewed. The duration of hospital stay prior to uterine evacuation ranged from less than an hour to 480 hours. All the patients received either prophylactic or therapeutic antibiotics. Use of parenteral antibiotics was significantly associated with severity of abortion, second trimester abortion, use of blood products and the interval between management’s decision and uterine evacuation. Uterine evacuation for retained products of conception was achieved by metallic curettage among 516 (83.4%) patients and by vacuum aspiration in 18 (2.9%). At all the study sites, Misoprostol or Oxytocin were used concurrently with surgical evacuation of the uterus. None use of analgesics or anesthetics in the four hospitals ranged between 12.4% to 28.8%.

**Conclusion:**

There is evidence of delayed patient care and prolonged hospital stay. Metallic curette was the primary method used for uterine evacuation across all the facilities. Pain management and antibiotics use was not standardized. A protocol has to be developed with the aim of standardizing post abortion care.

## Introduction

Globally an estimated 42 million abortions occur annually of which 20 million are unsafe. Complications of unsafe abortion lead to 70,000 maternal deaths and 5 million permanent or temporary maternal disabilities per year. Higher morbidity and mortality of women has been observed in the regions with restrictive abortion laws[1]. It is also noted that both cases of spontaneous and induced abortion present to health facilities as cases of spontaneous miscarriage for post abortion care [2, 3]. Traditionally, management of spontaneous abortion involved surgical evacuation of the uterus using dilatation and curettage for prevention of infection due to retained products of conception[4]. Progressively the use of manual or electrical vacuum suction has been widely recommended to replace dilatation and curettage for gestational age less than 13 weeks. This is because vacuum aspiration has fewer complications compared to metallic curettage and can be performed by mid-level health workers [5–7]. The World health organization (WHO) recommends the use of vacuum aspiration as the standard of care in the management of abortion of gestational age below 14weeks[8].

In settings where abortion is legal; it may not be difficult in differentiating spontaneous miscarriage from self-induced abortion and identifying those with risk of infection progressing to subsequent sepsis. In cases with less risk of infection, routine surgical evacuation of the uterus for miscarriage may not be justifiable, as it can pose a risk of introducing infection, cervical damage, uterine perforation, pain and hemorrhage. A Cochrane review of randomized clinical trials by James P Neilson et al revealed that medical treatment with misoprostol and expectant care (no intervention) are both acceptable alternatives to routine surgical evacuation provided that there are health service resources supporting all three approaches. Women having spontaneous abortions in the first trimester should be offered an informed choice on their plan of management[9]. Findings from a randomized controlled trial in Uganda revealed that vacuum aspiration and misoprostol are equally effective in complete evacuation of uterine contents in patients with incomplete abortion in the first trimester [10]

Evidence regarding post abortion care in Botswana is limited. A study conducted in Botswana reported that 11% of maternal deaths were due to pregnancy loss before 24 weeks[11]. Contraception prevalence rate of Botswana is 53% with high unmet needs. Poor utilization of contraceptives increases the risk of unintended pregnancy which may lead to unsafe abortion with a resultant significant burden to the health care system [12, 13]. This study aimed to assess the practice of post abortion care in selected hospitals in Botswana. The results will inform both clinicians and policy makers in improving post abortion care.

## Methodology

### Study setting

Botswana, a middle income country in Southern Africa, has a total population of 2.02 million inhabitants with a female to male ratio of 1.05. Half of this female population is in the reproductive age of 15–49 years with a total fertility rate of 2.9 Children per woman [14, 15]. There are 28 public hospitals: 3 referral, 10 district and 15 primary hospitals in the country. A 2012 report of referral Hospitals in Botswana revealed that abortion complications accounted for more than 60–65% of admissions to Gynecology wards (Unpublished report).

### Study design

A retrospective, institution based, cross sectional study was conducted at four selected hospitals in Botswana. Data was collected from patients`records admitted for the management of post-abortion complications between January and August 2014. The term post-abortion complication was used in our study for all patients admitted with pregnancy loss before 24 weeks regardless of the cause.

### Sampling method

Data was collected from four hospitals, selected using convenience sampling in the different districts. These included two referral: Princes Marina Hospital (PMH) and Nyangabgwe Referral Hospital (NRH); and two district hospitals: Mahalapye and Maun hospitals located in the southern, central and northern parts of the country. All patients’ records were reviewed for individual and clinical information, including their management outcome for post-abortion complications.

### Data collection

Data was collected by trained clinicians and midwives who were members of investigating team. The data collected included patient socio-demographic variables, abortion complications, patient management and treatment outcome. Information on patient management and outcomes such as: diagnosis, intervention interval and standard of care, use of blood products, operative interventions, means of uterine evacuation, administration of antibiotics, counseling, post abortion family planning and any abortion related maternal death were collected. Completeness and accuracy of the data were checked.

Data was collected at the respective hospitals using a structured data extraction sheet. All records of threatened abortion, ectopic pregnancy, molar pregnancy and other abnormal non pregnancy related uterine bleeding were excluded from the study. The patients were grouped into different levels of severity as follows:

**Low severity** (all criteria to be present): All recorded temperatures <37.2°C, no suspicious finding reported on evacuation, no sign of infection and no system or organ failure recorded in the file.
**Moderate severity:** Any recorded temperature 37.3–37.9°C or offensive smelling conception tissue or description of pelvic peritonitis recorded in the file.
**High severity:** Any recorded temperature > 38°C, any listing of organ or system failure, generalized peritonitis, septic shock, pulse>120 beats/minute, foreign body or sign of mechanical injury on evacuation.

### Data management and analysis

SPSS 22 software was used for data entry, cleaning and analysis. Frequencies, percentage, and chi-square test were utilized in the presentation of the findings. A p-value of less than 0.05 was considered statistically significant.

### Ethical issues

Ethical clearance was obtained from the institutional review boards of the University of Botswana, Ministry of Health and ethical committees of the four study hospitals. As the study was retrospective, patients had already been managed as per the hospital management protocol and hence the data extraction did not have any effect on the patient care. Since all the patients were not in the hospital at the time of data collection, informed consent was waived by the institutional review boards to access patient`s records.

## Result

The management of post-abortion complications among 619 patients who presented to the study sites was reviewed. The details of the background characteristics of the patients are described elsewhere [16]. At the time of inpatient care, 315 (50.9%) of the patients were reported to be HIV negative, 142 (22.9%) were HIV positive and 162 (26.2%) had an unknown HIV status.

A total of 550 (88.9%) patients required uterine evacuation using either surgical or medical means or both. Uterine evacuation was done using metallic curette in 516 (83.4%) of the patients and 161/516(31.2%) developed high or moderate severity complications. Vacuum aspiration was used in only 18 (2.9%) patients to evacuate retained products of conception of whom 4/18(22.2%) developed severe or moderate complications. Oxytocin and misoprostol were administered to 86 (13.9%) and 50 (8.1%) patients respectively in order to effect uterine evacuation concurrently with surgical means. Six (1%) patients were managed by laparotomy, 7 (1.1%) underwent hysterectomy and 4 (0.6%) had bowel surgery. A total of 10 (1.6%) patients were admitted to ICU. There were a total of 9 deaths; a case fatality rate of 1.5%. (Table 1). In almost three quarters of the patients, uterine evacuation was done under conscious sedation (Pethidine + Other Sedatives). General anesthesia was used in 15 (2.4%) patients, while ketamine alone was used in 15 (2.4%) patients (Table 2).

**Table 1:** Medical management, uterine evacuation and means of pain control in patient care.

Description	Frequency	Percentage
**Medical management**		
Antibiotic used as prophylaxis	273	44.1
Use of oral antibiotics	169	27.3
Use of parenteral antibiotics	177	28.6
IV fluids	239	38.6
Whole blood /PRBC	59	9.5
Fresh frozen plasma	8	1.3
Platelets	4	0.6
**Evacuation of uterus**		
Evacuation of the uterus	550	88.9
Re-evacuation of the uterus	19	3.1
**Pharmacological means of evacuation**		
Misoprostol	50	8.1
Oxytocin	86	13.9
Prostaglandin E2	14	2.3
**Surgical and other means of evacuation**		
Metallic curettage	516	83.4
Vacuum aspiration	18	2.9
Spontaneous expulsion	19	3.1
Other modes of expulsion	5	0.9
**Other procedures and care**		
Laparotomy	6	1.0
Hysterectomy	7	1.1
Bowel surgery	4	0.6
ICU admission	10	1.6

**Table 2:** Management of post-abortion complication across study hospitals.

Management type	PMH (%)	NRH (%)	Mahalapye (%)	Maun (%)
	N = 267	N = 170	N = 79	N = 103
**Antibiotics**				
parenteral	80 (30%)	49 (28.8%)	13 (16.5%)	35 (34%)
Oral	146 (54.7%)	5 (2.9%)	6 (7.6%)	12 (11.7%)
prophylactic	34 (12.7%)	121 (71.2%)	60 (75.9%)	58 (55.2%)
**Uterine evacuation**				
Metallic	204 (76.4%)	150 (88.2%)	76 (96.2%)	86 (83.5%)
Vacuum aspiration	10 (3.7%)	2 (1.2%)	0	6 (5.8%)
Misoprostol	39 (14.6%)	2 (1.2%)	2 (2.5%)	7 (6.8%)
Oxytocin	21 (7.9%)	0	32 (40.5%)	33 (32%)
**Anaesthesia and Analgesia**				
Not used	33 (12.4%)	49 (28.8%)	20 (25.3%)	15 (14.6%)
Pethidine and/or Diazepam	228 (85.4%)	114 (67.1%)	24 (30.4%)	85 (82.5%)
Spinal aesthesia	0	1 (0.6%)	1 (1.3%)	0
General aesthesia	3 (1.1%)	6 (3.5%)	3 (3.8%)	3 (2.9%)
Other	3 (1.1%)	0	31 (39.2%)	0
**Use of blood products**				
Red blood cell/whole blood	28 (10.5%)	13 (7.6%)	5 (6.3%)	13 (12.6%)
Plate late/fresh frozen plasma	4 (1.5%)	5 (2.9%)	3 (3.8%)	0

All of the patients were given some form of antibiotics either prophylactic (44.1%) or therapeutic (55.9%). One third of all the patients received parenteral antibiotics. Use of prophylactic antibiotics was less common in one referral hospital (12.7%) compared to the other three hospitals ranging between 55–76%. In Princess Marina Hospital (PMH), the use of misoprostol was higher (14.6%) compared to the other hospitals and oxytocin use was higher in district hospitals: Mahalapye (40.5%) and Maun (32%). Procedure without anesthesia and analgesia across the study hospitals ranges between 12.4% in PMH to 28.8% in Nyangabgwe Referral Hospital (NRH) (Table 2). Blood products were used in 9.5% of patients despite 14.9% of patients having a recorded hemoglobin level <7g/dl which is the level indicating need for transfusion in our setting. Further analysis on the use of blood and blood components is described in another article[17].

The duration of hospital stay before uterine evacuation ranged from less than an hour to 480 hours with a median of 12 hours. Patient hospital stay after uterine evacuation or other surgical intervention ranged from 1 to 456 hours with a median of 24 hours. One hundred and ninety eight (36.7%) patients stayed in hospital for more than 24 hours after uterine evacuation. The main reasons of staying over 24 hours were administration of parenteral antibiotics (25.9%) and transfusion of blood product (15.2%) (Table 3).

**Table 3:** Time duration in relation to the course of patient in post abortion care.

Time	Frequency	Percentage
**Duration of vaginal bleeding before presentation**		
≤ 3 days	390	63
3–5 days	161	26
> 5 days	65	10.5
Not specified	3	0.5
Range: 0–30 days	Median: 1day	
**Duration of Hospital stay before planed uterine evacuation, N = 518**		
≤ 24 hours	378	73
24–72 hours	99	19.1
>72 hours	41	7.9
Range: 0–480 hours	Median: 12 hours	
**Duration of hospital stay after evacuation of uterus, N = 539**		
≤ 24 hours	341	63.3
hours	139	25.8
≥72 hours	59	10.9
**Reasons for staying > 24 hours post uterine evacuation, N = 198**		
For antibiotics	51	25.9
For transfusion of blood product	30	15.2
Others	8	3.8
Unknown reason	109	55.1

Patients with severe complications of abortion were more likely to receive parenteral antibiotics compared to moderate and low complication (p< 0.001). Those patients admitted with second trimester abortion were more likely to receive parenteral antibiotics (p = 0.03) compared to those with first trimester loss. Transfusion of any of blood products had a strong association with administration of parenteral antibiotics (P<0.001). Use of misoprostol or oxytocin was significantly related with hospital stay of more than 24 hours (p = 0.003) and is marginally associated with second trimester pregnancy loss (P = 0.06). Use of sharp metallic curette was consistently high across study hospitals (P<0.001) and its use in the first trimester was slightly higher than second trimester (p = 0.069)(Table 4).

**Table 4:** Relationship of selected variables with parenteral antibiotics use and means of uterine evacuation.

Variable		Parenteral Antibiotic		Chi-square (p-value)
		Yes	No	
		N(Row%)	N(Row%)	
Severity of abortion	Severe	54(79.40%)	14(20.60%)	182.21 (0.00)
	Moderate	69(55.20%)	56(44.80%)	
	Mild	54(12.70%)	372(87.30%)	
Any transfusion binary	yes	39(60.90%)	25(39.10%)	36.57 (0.00)
	No	138(24.90%)	417(75.10%)	
GA by trimester	1st trimester	84(24.90%)	253(75.10%)	4.71 (0.03)
	2nd trimester	71(33.50%)	141(66.50%)	
Hours before evacuation category	Up to 24 hours	146(30.5%)	333(69.5%)	3.69 (0.055)
	Over 24 hours	31(22.1%)	109(77.9%)	
		Metallic curette		
		Yes	No	
Hospital	PMH	204(76.4%)	63(23.6%)	21.62 (0.00)
	NRH	150(88.2%)	20(11.8%)	
	Mahalapye	76(96.2%)	3(3.8%)	
	Maun	86(83.5%)	17(16.5%)	
GA by trimester	1st trimester	290(86.10%)	47(13.90%)	3.29 (0.069)
	2nd trimester	170(80.20%)	42(19.8%)	
		Misoprostol/Oxytocin		
		Yes	No	
GA by trimester	1st trimester	62(18.4%)	275(81.60%)	3.43 (0.06)
	2nd trimester	53(25%)	159(75.00%)	
Hospital stay in 24 hours	up to 24 hours	88(18.4%)	391(81.6%)	8.83 (0.003)
	Over 24 hours	42(30%)	98(70%)	

Almost half of patients reported using some form of contraceptive at the time of conception of the pregnancy and condom was the most used method (38.3%). Only 20% of the patients were recorded as having undergone post abortion counseling and less than 5% were recorded as having discussed some form of contraceptive method on discharge.

## Discussion

All the patients had received either prophylactic or therapeutic dose of antibiotics. There was preferential use of metallic curettage for uterine evacuation compared to vacuum aspiration across all the four hospitals. A significant proportion of patients were admitted as referrals. Similarly, significant number of patients had delayed intervention. This may have had a negative impact on patient outcomes such as the high case fatality rate and the protracted hospital stay of more than 24 hours observed in 36.7% of patients. The patient care in terms of uterine evacuation, pain management, antibiotic use and admission to intervention interval was neither standardized nor uniform. This highlights a critical need to develop guidelines and protocols to ensure appropriate patient management across hospitals in Botswana.

Twenty three percent of the patients were HIV positive and more than a quarter of the study patients`HIV status was not known at the time of discharge. This needs to be addressed as part of the elements of post abortion care and should be used as opportunity for HIV screening and prevention. As it was reported from our previous work, there was a high incidence of second trimester abortion[16]. Such second trimester post abortion care is known to be more demanding in terms of resources because of increased maternal near miss or death according to WHO multicenter study[18]. In some patients, the time duration before uterine evacuation extended to 480 hours. This is likely due to unnecessary admission of patients with threatened abortion to the Gynecology ward which subsequently progressed to inevitable abortion. From this study, we found that 36% of patients had hospital stay of more than 24 hours after uterine evacuation of products of conception for either blood transfusion, parenteral antibiotics or the management of other complications. Obviously the cost of inpatient care is much more expensive than outpatient care and this finding indicates that post abortion care imposes a significant burden on the health care service of the country which needs further investigation. Estimated cost of post abortion care per patient in Africa is US$83. In Ethiopia the annual direct and indirect cost was estimated to be US $47 million[19, 20].

All patients received some form of antibiotic, more than half (55.9%) of the patients got a therapeutic course while 44.1% were given a prophylactic dose. This rate of antibiotic usage appears higher than most other studies in Africa. In South Africa, antibiotic usage as part of abortion management decreased from 44.3% in 1994 to 33.5% in 2000[21, 22]. In Ethiopia antibiotics have been reported to be used in 83% of the cases[23]. However it is not known whether results from these studies included prophylactic antibiotics or only assessed use of therapeutic courses. WHO recommends universal use of a single dose of prophylactic antibiotics for all women having surgically induced abortion regardless of risk of developing pelvic inflammatory disease[8]. With regard to antibiotic use in cases of incomplete abortion, there is insufficient evidence for the universal use of prophylactic antibiotics to date[24]. In our study, the routine use of antibiotics might have been influenced by two important factors: Firstly, almost all the patients were admitted as spontaneous post abortion complication in a setting where it was not easy to differentiate a spontaneous abortion from a self-induced clandestine abortion; and secondly the high prevalence of HIV infection. The Botswana safe motherhood guideline recommends the universal use of antibiotics in the management of abortions with the exception of missed and threatened abortions[25].

With regard to uterine evacuation, sharp metallic curettage was used in 83% of the patients and vacuum aspiration was used in 2.9% of the patients. This preference of metallic curette was found to be similar along all the study hospitals. These findings are consistent with the findings from a South African study that revealed a vacuum aspiration rate of 2.8% in 1994 and 14.4% in 2000 in tertiary level hospitals in contrast with the use of sharp curettage at 82% [21, 22]. The practice of vacuum aspiration in our study is much lower than the study in Ethiopia where 68% used vacuum aspiration for abortions at gestational age below 12 weeks and another study in Kenya with 65% use of vacuum aspiration in eligible cases [26]. A multi-national survey revealed that metallic curette was used predominantly in countries where abortion is restricted[18]. WHO recommends the use of either manual or electrical vacuum aspiration or misoprostol for incomplete abortion with uterine size less than or equal to 13 weeks[8]. Although electrical vacuum aspiration instruments are available in the Botswana health system, their use is mainly limited to the management of molar pregnancy and endometrial biopsy in tertiary hospitals by specialists. There is much evidence that vacuum aspiration can be safely used by mid-level health care providers and can be used widely including primary health care facilities with a proper referral system for complication management[27, 28].

In our study both Oxytocin and Misoprostol were used in13.1% and 8.1% respectively and mostly used concurrently with surgical means of uterine evacuation. There was higher use of Misoprostol in PMH while district hospitals were using more of Oxytocin. This may be due to non-availability of the medications or difference in the preference of attending doctors which requires further study to understand the situation very well. According to evidence from a randomized control trial from Uganda and recommendations from WHO, there is significant evidence that misoprostol can be used singly as an alternative option of uterine evacuation method in incomplete abortion [8, 10]. Evidence has also shown that there is no difference in treatment outcomes between conservative expectant management, use of misoprostol and surgical(vacuum aspiration) management of incomplete miscarriage of pregnancy less than 13 weeks [9].

There was a high case fatality rate of 1.5% in this study. According to the United Nations process indicators of standard emergency obstetric care, the case fatality rate due to direct obstetric complications should not exceed 1% [29]. Availing quality emergency obstetric care, minimizing unmet need of contraception and legalization of abortion are believed to be fundamental factors in averting maternal disability and mortality related with abortion complications [30, 31].

## Limitation of the study

This study being retrospective in nature and due to incompleteness of information, limited our ability to analyze triage to admission time and decision of management to intervention time which could have had crucial impact on patient management outcomes. Our study did not assess the practice of manual or electrical vacuum aspiration by mid-level and junior health care providers. The type of antibiotics used in the management of post abortion complications and the place of procurement of self-induced abortion by the patient is not addressed in this study and these are areas for future investigations.

## Conclusion

This study has revealed that there was a delay in initiation of critical emergency post abortion care such as uterine evacuation. When curettage is performed, there was a preference of metallic curette over vacuum aspiration. Pain management and use of antibiotics was not standardized. Standardizing patient management protocols in terms of uterine evacuation, antibiotics use and pain management is mandatory. The use of WHO recommended less invasive vacuum aspiration or medical means of uterine evacuation should be strengthened. Decentralization of post abortion care services is recommended in order to avoid delayed initiation of patient care. A prospective study with the objective of investigating the process of triage to admission and intervention is strongly recommended. Further investigation of health care providers’ experience towards use of manual or electrical vacuum aspiration in the management of post abortion complications is also recommended. It is important to strengthen incorporate post abortion care training into the curriculum of medical professionals in Botswana.

## References

[1] Shah I, Ahman E. Unsafe abortion: global and regional incidence, trends, consequences, and challenges. J Obstet Gynaecol Can. 2009;31(12):1149–58. 20085681

[2] Sedgh G, Henshaw S, Singh S, Åhman E, Shah IH. Induced abortion: estimated rates and trends worldwide. The Lancet. 2007;370(9595):1338–45. 10.1016/S0140-6736(07)61575-X 17933648

[3] Haddad LB, Nour NM. Unsafe abortion: unnecessary maternal mortality. Reviews in obstetrics and gynecology. 2009;2(2):122 19609407 PMC2709326

[4] Griebel CP, Halvorsen J, Golemon TB, Day AA. Management of spontaneous abortion. Am Fam Physician. 2005;72(7):1243–50. 16225027

[5] Lukman H, Pogharian D. Management of incomplete abortion with manual vacuum aspiration in comparison to sharp metallic curette in an Ethiopian setting. East African medical journal. 1996;73(9):598–603. 8991242

[6] Hemlin J, Möller B. Manual vacuum aspiration, a safe and effective alternative in early pregnancy termination. Acta obstetricia et gynecologica Scandinavica. 2001;80(6):563–7. 11380295

[7] Ansari R, Rathore S, Mustafa B. Manual Vacuum Aspiration: A Safe and Effective Alternative for the Surgical Management of Early Pregnancy Loss. Annals of Abbasi Shaheed Hospital & Karachi Medical & Dental College. 2014;19(1).

[8] WHO. Safe abortion:technical and policy guidance for health systems Second edition 2012;second Edition. 23700650

[9] Neilson JP, Gyte GM, Hickey M, Vazquez JC, Dou L. Medical treatments for incomplete miscarriage (less than 24 weeks). The Cochrane Library. 2010. 10.1002/14651858.CD007223.pub2 PMC4042279 20091626

[10] Weeks A, Alia G, Blum J, Winikoff B, Ekwaru P, Durocher J, et al A randomized trial of misoprostol compared with manual vacuum aspiration for incomplete abortion. Obstetrics & Gynecology. 2005;106(3):540–7. 16135584 10.1097/01.AOG.0000173799.82687.dc

[11] Ray S, Madzimbamuto F, Ramagola-Masire D, Phillips R, Mogobe K, Haverkamp M, et al Review of causes of maternal deaths in Botswana in 2010. SAMJ: South African Medical Journal. 2013;103(8):537–42. doi: 10.7196/samj.6723 23885735 10.7196/samj.6723

[12] WHO. World heath statistics. 2013.

[13] Mogobe KD, Tshiamo W, Bowelo M. Monitoring maternity mortality in Botswana. Reproductive Health Matters. 2007;15(30):163–71. doi: 10.1016/S0968-8080(07)30330-3 17938081 10.1016/S0968-8080(07)30330-3

[14] CSO. Botswana Population and Housing Census. 2011.

[15] /UNFPA WU. WHO/UNICEF/UNFPA/World Bank MMR report. 2010 2010. Report No.

[16] Melese T, Habte D, Tsima BM, Mogobe KD, Chabaesele K, Rankgoane G, et al High Levels of Post-Abortion Complication in a Setting Where Abortion Service Is Not Legalized. PloS one. 2017;12(1):e0166287 doi: 10.1371/journal.pone.0166287 28060817 10.1371/journal.pone.0166287 PMC5217963

[17] Tsima B, Melese T, Mogobe K, Chabaesele K, Rankgoane G, Nassali M, et al Clinical use of blood and blood components in post-abortion care in Botswana Transfusion Medicine. 2016. 10.1111/tme.12320 27214516

[18] Dragoman M, Sheldon W, Qureshi Z, Blum J, Winikoff B, Ganatra B. Overview of abortion cases with severe maternal outcomes in the WHO Multicountry Survey on Maternal and Newborn Health: a descriptive analysis. BJOG: An International Journal of Obstetrics & Gynaecology. 2014;121(s1):25–31. 24641532 10.1111/1471-0528.12689

[19] Vlassoff M, Walker D, Shearer J, Newlands D, Singh S. Estimates of health care system costs of unsafe abortion in Africa and Latin America. International Perspectives on Sexual & Reproductive Health. 2009;35(3). 10.1363/ipsrh.35.114.09 19805016

[20] Vlassoff M, Fetters T, Kumbi S, Singh S. The health system cost of postabortion care in Ethiopia. International Journal of Gynecology & Obstetrics. 2012;118:S127–S33. 22920616 10.1016/S0020-7292(12)60011-3

[21] Fawcus S, McIntyre J, Jewkes R, Rees H, Katzenellenbogen J, Shabodien R, et al Management of incomplete abortions at South African public hospitals. 1997. 9254786

[22] Brown H, Jewkes R, Levin J, Dickson-Tetteh K, Rees H. Management of incomplete abortion in South African public hospitals. BJOG: An International Journal of Obstetrics & Gynaecology. 2003;110(4):371–7. 12699798

[23] Gebreselassie H, Fetters T, Singh S, Abdella A, Gebrehiwot Y, Tesfaye S, et al Caring for women with abortion complications in Ethiopia: national estimates and future implications. International perspectives on sexual and reproductive health. 2010:6–15. doi: 10.1363/ipsrh.36.006.10 20403801 10.1363/ipsrh.36.006.10

[24] May W, Gülmezoglu A, Ba-Thike K. Antibiotics for incomplete abortion. Cochrane Database Syst Rev. 2007;4. 10.1002/14651858.CD001779.pub2 PMC12013248 17943756

[25] botswana MoH. The safe motherhood Initiative (SMI) Guidlines for Antenatal Care and the management of Obstetric Emergencies and Prevention of Mother to Child Transmission of HIV. Botswana 2010 p. 116.

[26] Izugbara C, Kimani E, Mutua M, Mohamed S, Ziraba A, Egesa C. Incidence and Complications of Unsafe Abortion in Kenya: Key Findings of a National Study. Nairobi, Kenya: African Population and Health Research Center, Ministry of Health, Kenya, Ipas, and Guttmacher Institute. 2013.

[27] Dickson-Tetteh K, Billings DL. Abortion care services provided by registered midwives in South Africa. International Family Planning Perspectives. 2002:144–50.

[28] Warriner IK, Meirik O, Hoffman M, Morroni C, Harries J, Huong NM, et al Rates of complication in first-trimester manual vacuum aspiration abortion done by doctors and mid-level providers in South Africa and Vietnam: a randomised controlled equivalence trial. The Lancet. 2006;368(9551):1965–72. 10.1016/S0140-6736(06)69742-0 17141703

[29] Paxton A, Bailey P, Lobis S. The United Nations Process Indicators for emergency obstetric care: reflections based on a decade of experience. International Journal of Gynecology & Obstetrics. 2006;95(2):192–208. 17074557 10.1016/j.ijgo.2006.08.009

[30] Hord C, David HP, Donnay F, Wolf M. Reproductive health in Romania: reversing the Ceausescu legacy. Studies in family planning. 1991:231–40. 1949105

[31] Stephenson P, Wagner M, Badea M, Serbanescu F. Commentary: the public health consequences of restricted induced abortion—lessons from Romania. American Journal of Public Health. 1992;82(10):1328–31. 1415854 10.2105/ajph.82.10.1328 PMC1695851

